# Comparison of ohmic heating‐ and microwave‐assisted extraction techniques for avocado leaves valorization: Optimization and impact on the phenolic compounds and bioactivities

**DOI:** 10.1002/fsn3.3556

**Published:** 2023-07-06

**Authors:** Lale Gumustepe, Nevriye Kurt, Ebru Aydın, Gulcan Ozkan

**Affiliations:** ^1^ Department of Food Engineering, Faculty of Engineering Suleyman Demirel University Isparta Turkey

**Keywords:** antidiabetic, antioxidant, avocado leaves, green extraction, pruning waste, RSM

## Abstract

Avocado tree pruning activities generate a substantial amount of residual biomass, which includes different parts of the plant, such as leaves, twigs, branches, and small fruits. This study aimed to investigate the impact of different green extraction methods of microwave‐assisted extraction (MAE) and ohmic heating‐assisted extraction (OHAE) for the phenolic extraction of avocado leaves based on a statistical approach, central composite design (CCD), and response surface methodology (RSM). Water was preferred using as an environmentally and health‐friendly solvent for both methods. The phenolic composition, antioxidant activity, and antidiabetic potential of the extracts were identified and comparatively assessed. The developed models exhibited a high degree of reliability with optimal conditions for OHAE and MAE, which were determined as 9.38 V/cm voltage gradient, 6 min extraction time, at 60°C, 5 min, and 1 g dried leaf/100 mL water. Epicatechin was identified as the primary phenolic compound in OHAE extracts, while chlorogenic acid was the dominant compound in MAE extracts. The extracts obtained from OHAE and MAE were tested for their ability to inhibit α‐glucosidase activity, with IC_50_ (mg/mL) values of 0.85 and 1.14, respectively. The DPPH radicals scavenging activity (IC_50_ mg/L) of OHAE and MAE were detected as 2.96 and 3.41, respectively. In conclusion, both methods yielded extracts rich in polyphenols that displayed high antioxidant activity, but OHAE was found to be superior to MAE in terms of TPC, DPPH, and antidiabetic activities. The results of this study have the potential to make significant contributions toward promoting the principles of a circular economy by facilitating the valorization of the avocado pruning waste.

## Graphical abstract

The paper addresses the hypothesis that using green extraction techniques may provide time, cost, and energy saving compared to traditional methods. We show that using ohmic (OHAE)‐ and microwave‐assisted extraction (MAE) techniques for Fuerte avocado pruning waste leaves phenolics provides less solvent consumption and better extraction efficiency with a short extraction time and low energy consumption.

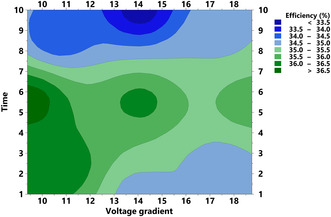

## 1 INTRODUCTION

Interest in biologically active natural products is rising, as concerns about ecological and environmentally friendly increase attention under the “zero waste” strategy. Therefore, a new approach is getting attention as an alternative and low‐cost source of several functional components of interest for many sectors, including the food industry. Avocado, commonly referred to as “alligator pear,” is classified under the *Plantae* kingdom, Lauraceae family, Laurales order, *Persea* genus, and *Persea americana* species (Adeyemi et al., 2002). FAOSTAT (2021) reported that global avocado production exceeded 8 million tons in the cultivation area of 858,152 hectares (FAOSTAT, 2021). In addition, both the fruit and by‐products of avocado are important for the food market, cosmetics, and pharmaceuticals worldwide and therefore it is a considerable source of commercial income. Avocado trees require pruning during both winter and summer seasons, which involves removing unproductive branches to increase light penetration into the lower canopy during summer and promote fruit production (Menzel & Le Lagadec, 2014). Therefore, the pruning biomass needs to be considered to create value‐added products that can benefit both the environment and the economy. A significant quantity of avocado tree residual biomass generated from pruning activities was measured to be between 780 and 1245 kg/ha of biomass on an annual basis, based on a standard of 100 trees per hectare, when measured on a dry weight basis (Soria‐González et al., 2022; Tauro et al., 2022). The literature indicated that avocado fruit, leaves, seed, and peel are reported to contain important resources of various bioactive compounds (Castro‐López et al., 2019; Gümüştepe et al., 2022). In different studies, the antioxidant activity of different parts of avocado was compared based on their bioactive compound content. These studies demonstrated that the antioxidant activity from the highest to the lowest of the avocado morphological parts is in order of leaves > peel > pulp oil > pulp (Kumar & Cumbal, 2016). Thus, avocado leaves represent a valuable source for obtaining value‐added compounds that possess potent antioxidant activity, such as chlorogenic acid, epicatechin, luteolin, quercetin, and rutin (Gümüştepe et al., 2022) and may have a potential application for its health‐promoting activities such as cancer, cardiovascular diseases, antidiabetic, anti‐inflammatory, antiviral, antifungal, and antibacterial activities (Kumar et al., 2017; Makopa et al., 2020).

The chosen solvent type's impact on humans and the environment are also important to provide nontoxicity in the obtained products, to make them healthier, more economical, and eco‐friendly. Various solvents (hydrochloric acid, petroleum ether, methanol, ethanol, and/or water) were applied using conventional extraction techniques (maceration and infusion) to extract bioactive compounds from avocado leaves (Polat Kose et al., 2020; Rivai et al., 2019) in the literature. In the current study, water was utilized for phenolic extraction as an environmentally and health‐friendly solvent. Besides, extraction parameters and techniques applied are important steps of the extraction process. Nowadays, new extraction techniques known as green technologies (microwave, ultrasound, high pressure, supercritical fluid, ohmic, etc.) are also used. The most important reasons for the preference of these new techniques are due to their environmental benefit as well as the increased efficiency of the extracted component (Rodrigues et al., 2019). Only one advanced technique consistent with the principles of green chemistry was used to extract avocado leaf phenolics in the literature, and this technique involved ultrasound‐assisted extraction (Castro‐López et al., 2019; Che‐Galicia et al., 2020). However, this is the first study that compares the effect of different green technology (OHAE and MAE techniques) on avocado leaves phenolics aqueous extraction optimization. Both OHAE (Joule heating or electrical resistance heating) and MAE are modern green extraction techniques and are used extensively in many areas. OHAE enables better extraction of bioactive components by causing electroporation in cellular tissues due to the applied electric field (Pereira et al., 2021). Also, different OH studies reported the improved phenolic extraction efficiency of polyphenols such as red grape pulp, algae (*Gracilaria vermiculophylla*), sugar grass (*Stevia rebaudiana*), vine, and yacon (black apple) leaves, colored potato, and anthocyanins from black rice bran (Khajehei et al., 2017; Moongngarm et al., 2022; Pereira et al., 2021). The principle of MAE is based on the polarization of polar water molecules in food by the electric field, whereas this vibration of molecules occurs in heat which then increases extraction efficiency and decreases extraction time. Besides, these conditions decrease the rate of degradation of phenolic substances and other bioactive components (Álvarez et al., 2017; Barba et al., 2016).

The consumption of avocados is increasing worldwide, owing to the presence of health‐promoting bioactive compounds. However, this demand is leading to a substantial increase in agricultural waste generated globally. In response, various technologies for extracting bioactive compounds from this waste and utilizing them as functional food and nutraceutical ingredients are needed to consider in terms of sustainability and circular economy. In this study, optimization of the phenolic extraction was conducted based on a mathematical model central composite design (CCD) and response surface methodology (RSM) to understand their optimal extraction conditions and design an optimal process as they are rapid and require less time and material (Borkowski, 1995; Khuri & Mukhopadhyay, 2010). To the best of our knowledge, this research is the first report regarding the comparison of two methods (OHAE and MAE) for the optimization of bioactive components from Fuerte variety avocado leaves using the statistical method RSM and CCD. In addition, both extracts' antioxidant and antidiabetic activities were analyzed and compared for the first time to indicate the effect of the extraction technique on the biofunctional properties.

## 2 MATERIALS AND METHODS

### 2.1 Materials

The leaves of the Fuerte avocado variety tree were obtained as research material from a 7000 m^2^ orchard where Fuerte, Hass, Pinkerton, and Bacon avocado varieties were planted in full efficiency and 6 × 6 m planting distance in coordinates: 36°15′28.5″N 32°19′59.2″E, Gazipaşa District of Antalya. The preparation process of the leaves included phases of cleaning, sifting, and drying in an air dryer (Mikrotest, Ankara, Turkey) at 60°C until achieving a moisture content of 3.91%. Following that the dried fruits were ground to obtain the fine powder, using a laboratory‐scale grinder (Waring Commercial Laboratory Blender, USA). Next, the samples were sifted and passed through a sieve (Kocintok, Turkey) with 500 μm mesh and were tightly packed in polyethylene bags to store at −45°C for further experiments.

### 2.2 Experimental design: Optimization of microwave‐ and ohmic heating‐assisted extractions (MAE and OHAE) parameters

RSM and CCD were used for determining optimal OHAE (Eraktek İnovasyon, Konya, Turkey) and MAE (Millipore, Italy) conditions of dried avocado leave phenolics using Minitab Statistical Software (Minitab 20.0). Preliminary experiments were conducted to determine the independent variables based on the literature review for both methods. The effect of two dependent variables (extraction efficiency and TPC) was used to detect optimum conditions of phenolic extraction. In this study, water was used as a solvent for OHAE and MAE methods. All runs were randomized in order and three experimental responses are presented (TPC, DPPH free radical scavenging activity, and extraction efficiency) (Radojković et al., 2012). Model adequacy and regression tests were evaluated by *R* ^2^ and adjusted *R* ^2^. To obtain the regression coefficients (*β*), the experimental data were fitted to a second‐order polynomial model. For the RSM analysis, the extended second‐order polynomial model *Z* = $\beta_{0}+\sum_{i=1}^{2}\beta_{i}X_{i}+\sum_{i=1}^{3}\beta_{\mathrm{ii}}X_{i}^{2}+\sum_{i=1}^{2}\sum_{j=i+1}^{2}\beta_{\mathrm{ii}}X_{i}X_{j}$ was employed. Here, *Z* represents the dependent variable, *X* represents the independent variable, and the constant coefficients are defined as *β*_0_ for the intercept, *β*_*i*_ for linear, *β*_*ii*_ for quadratic, and *β*_*ij*_ for the two‐factor interaction coefficient.

#### 2.2.1 OHAE parameters

The OHAE equipment consisted of electrodes, an ohmic cell, a power supply, and monitoring devices for current, voltage gradient, and resistance. The power supply had a maximum output of approximately 4 kW and allowed manual adjustment of the voltage output within the range 0–400 V. It generated alternating current with a maximum of 10 A at a frequency of 50–60 Hz. The ohmic cell, made of glass, had dimensions of 10 × 10 × 20 cm^3^, enabling periodic visualization of any changes occurring during the extraction process. Stainless steel electrodes with dimensions of 6 × 10 cm^2^ were used in the setup. The distance between the electrodes within the ohmic cell was maintained at 10 cm. To monitor the temperature of the extraction medium, a Teflon‐coated thermocouple was employed in the center of the ohmic cell. It was assumed that the temperature distribution within the heating volume was uniform. To maintain stability, a thermostat was utilized, providing on/off control with a temperature oscillation of ±1°C. During the experiments, different voltage gradients were applied based on the response surface methodology (RSM). For each experiment, the current and temperature were recorded, and photographs of the ohmic cell were taken at 5‐s intervals. Based on the results of preliminary experiments, the temperature detected was 95°C, NaCl/water was 0.15% (w/v), and solid/solvent ratio was found to be 1.00% (w/v). The incorporation of salt into an extraction medium has been found to enhance its electrical conductivity, facilitating the generation of heat within the mixture (Khajehei et al., 2017). Thus, prior to the implementation of OHAE in this study, a concentration of 0.15% w/v sodium chloride (NaCl) was introduced into the mixture of leaves and water. The amount of NaCl was determined based on the preliminary study. This addition was aimed at improving the electrical conductivity of the extraction medium based on providing a shorter extraction time. In addition, independent variables were determined as a voltage gradient (*X*_1_: 10.75–18.75 V) and extraction time (*X*_2_: 1–10 min). A central composite design was applied with 5 center points and consisted of 13 experiments and its TPC and extraction efficiency were measured as the response. Three replicates were performed in each extraction for the analysis of responses. Table 1 presents the RSM design of experiments and the selected ranges.

**TABLE 1:** Central composite design and experimental matrix.

Run order	Ohmic‐assisted extraction				Microwave‐assisted extraction			
	Voltage gradient	Time	Efficiency	TPC	Solid/solvent	Temperature	Efficiency	TPC
1	17.38	2.32	34.65	28650.27	1.00	45.00	16.74	13459.01
2	14.07	1.00	34.71	26118.08	4.25	45.00	15.82	10692.12
3	14.07	5.50	36.02	30769.21	1.95	34.39	15.64	12722.24
4	10.75	8.68	34.08	31737.82	4.25	45.00	15.82	10666.98
5	14.07	5.50	36.19	30611.75	6.55	34.39	14.25	9005.55
6	17.38	8.68	34.89	24223.75	4.25	30.00	14.49	10157.30
7	10.75	2.32	36.49	26405.96	4.25	45.00	15.78	10667.73
8	14.07	5.50	36.46	30605.47	7.50	45.00	14.84	10386.63
9	18.75	5.50	35.83	27211.69	4.25	45.00	15.78	10667.98
10	14.07	10.00	33.22	26887.77	1.95	55.61	17.14	12533.73
11	14.07	5.50	36.06	30657.00	6.55	55.61	15.70	11968.88
12	9.38	5.50	36.89	31036.83	4.25	45.00	15.80	10730.55
13	14.07	5.50	36.22	30597.40	4.25	60.00	16.60	12264.51

*Note*: Voltage gradient (V/cm), time (min), solid solvent ratio (g/100 mL), temperature (°C), efficiency (%), and total phenolic content (mg GAE/100 g extract—TPC).

#### 2.2.2 MAE parameters

Preliminary experiments were conducted to determine the independent variables based on the literature review. Consequently, different ranges of temperature (°C), extraction time (min), microwave power (W), and solid/solvent ratio (g/100 mL) were analyzed as independent variables, and TPC and extraction efficiency were chosen as responses of these independent variables. The solid/solvent ratio ranged from 0.5 to 10 g/100 mL, microwave power varied between 200 and 500 W, while the time and temperature were set between 2 and 20 min and 25–70°C, respectively. Based on the results of the preliminary experiments, the microwave power and time were constant at 500 W and 5 min, respectively, and the independent variables were determined as the solid/solvent ratio (*X*_1_) and temperature (*X*_2_). In addition, the minimum and maximum factor levels of temperature (30–60°C) and solid/solvent ratio (1.0–7.5 g/100 mL) were detected as independent variables. Similar to OHAE extraction, a central composite design was applied with 5 center points and consisted of 13 experiments, and its TPC and extraction efficiency were measured as the responses. Table 1 presents the RSM design of experiments and the selected ranges.

In addition, hot maceration was used as a traditional extraction method for comparison purposes to green extraction techniques. Grounded avocado leaves (1 g) were extracted in 100 mL water at 95°C for 120 min and then their TPC and extraction efficiency were measured as a response (Demircan et al., 2023).

#### 2.2.3 Extraction efficiency

The extracts obtained by CCD were centrifuged (Sigma, Germany), and 5 mL of the supernatant was dried in incubators at 105°C until the weight became constant. The efficiency was quantified by measuring the amount of dry leaves in grams per 100 g of extract. Each experiment had three replicates.

#### 2.2.4 Total phenolic content (TPC)

The extracts obtained by CCD were centrifuged and filtered. The TPC method utilized in this study was directly adopted from the methodology established by Ceylan et al. (2023). The TPC calculations were conducted utilizing a calibration curve generated using gallic acid as a reference standard. The TPC values were expressed as milligrams of gallic acid equivalent (GAE) per gram of gram extract. For each sample, three replicates were done.

### 2.3 Determination of phenolic constituents by HPLC‐DAD analysis

RP‐HPLC (Shimadzu Scientific Instruments, Tokyo, Japan) with a diode array detector was used to analyze the phenolic constituents in the samples. The method for the detection of phenolic compounds by HPLC‐DAD utilized in this study was directly adopted from the methodology established by Ceylan et al. (2023). Identification and quantification were done by comparing the samples with standards, and the phenolic components in the samples were expressed as mg/100 g extract. Three replicates were performed for each sample.

### 2.4 DPPH radical scavenging activity

The DPPH activities of avocado leaves phenolic extracts were determined after centrifugation and filtration of it. The DPPH method utilized in this study was directly adopted from the methodology established by Ceylan et al. (2023). The IC_50_ value of the extract concentration, to inhibit 50% of the free radicals, was determined based on the graph produced from DPPH (%) activity, and the results were shown as IC_50_ = mg/mL. All analyses were performed in triplicate.

### 2.5 Antidiabetic activity

The presence of α‐glucosidase enzyme activity was detected in rat intestinal powder. To optimize the assay conditions, the Michaelis constant (*K*_m_/*V*_max_) was determined (Aydin, 2015). The Km value was found to be 18 mM, and the Vmax value was determined as 0.09 μmol sucrose hydrolysis per minute. Different concentrations of the enzyme and varying incubation times at 37°C were tested to identify the optimal incubation time and enzyme concentration. Based on preliminary studies, a mixture of 18 mM sucrose (200 μL) and 15 mg/mL acetone rat intestinal powder (200 μL) was prepared along with 100 μL of sodium phosphate buffer (10 mM, pH 7.0) as the control sample. To assess the antidiabetic activity of the extract, 100 μL of sodium phosphate buffer was replaced with 100 μL of the extract/solution and incubated for 15 min at 37°C. Enzyme activity was halted by adding 750 μL of acetone to the mixtures, followed by 10 s of vortexing. Subsequently, the acetone was evaporated under nitrogen gas, and the supernatant was filtered. The quantification of sucrose and its products (glucose and fructose) was performed using an HPLC system equipped with a refractive index detector (RID) (Shimadzu, Japan).

### 2.6 Statistical analyses

The data are presented as means with their corresponding standard deviations. Statistical analysis was conducted using SPSS 20.0, and Duncan's multiple‐comparison test was performed to assess the significance of the treatment differences.

## 3 RESULTS AND DISCUSSION

Avocado fruits are a significant cash crop worldwide with 858,152 hectares in 2021 (FAOSTAT, 2021). Therefore, the tremendous quantity of accumulation of avocado pruning waste is inevitable. This is the first comparative research about the utilization of OHAE and MAE applied for polyphenols recovery from the waste generated by pruning the Fuerte variety of avocado leaves. In addition, antioxidant and antidiabetic activities of optimized extraction were detected and compared with the chemical drug acarbose.

### 3.1 Extraction modeling of the efficiency and total phenolic content

Optimizing the extraction processes to enhance the amount of extracted responses is essential. The CCD was used to examine the relationship between the characteristics of two extraction methods (OHAE and MAE) and the factors that affect them. Two independent variables were monitored in this study. Voltage and time were independent variables of OHAE, whereas solid/solvent ratio and temperature were the independent variables of MAE. Besides, the dependent variables of each method were the same (TPC and extraction efficiency). The optimization process involved conducting 13 runs for each extraction method, and the independent and dependent variables are provided in Table 1. The main objective was to maximize the responses to obtain higher extraction efficiency and total phenolic content.

According to Table 1, it is determined that the extract efficiency (%) obtained by OHAE of the leaves is in the range of 33.22%–36.89%, whereas it was between 14.25% and 17.14% for MAE. The highest extract efficiency was found in the OHAE techniques with a voltage value of 9.38 V/cm and an application time of 5.5 min (run 12), while the lowest efficiency was detected in run 10 with a voltage value of 14.07 V/cm and extraction time of 10 min. For comparison with the MAE method; the lowest extraction efficiency was obtained in run 5 for 6.55 g solid/solvent ratio and 34°C and the highest extract efficiency was found with a solid/solvent ratio of 1.95 g and temperature of 56°C for run 10. Overall, the results suggest that OHAE is a promising method for extracting phenolic compounds from avocado leaves, with a higher efficiency than MAE. Also, for comparison with the traditional method, the extraction efficiency of avocado leaves phenolics applied with hot maceration conditions at 95°C and 120 min had an extract efficiency of 13.38%. The findings of this study suggest that OHAE and MAE can be a preferable alternative to traditional extraction methods, as it results in a higher extract efficiency of phenolic compounds from avocado leaves and lower energy consumption in terms of shorter extraction time and higher extraction efficiency. In addition, the 36.89% extract efficiency obtained by OHAE of avocado leaves' phenolics was higher than the results obtained by the MAE and traditional extraction method. In a previous study, it was also reported that the extract efficiency (%) obtained by utilization of water, ethanol, and methanol solvents using the traditional extraction method were 20.9, 16.8, and 18.6, respectively (Kamagate et al., 2016).

Table 1 also indicates the TPC (mg GAE/100 g extract) of the Fuerte variety avocado leaves and it was shown that the TPC values ranged from 24223.7 to 31737.8 for OHAE while the MAE application has a TPC value between 9005.5 and 13459.0. The highest amount of phenolic compounds was obtained under the extraction conditions of 10.75 V/cm voltage and 8.68 min extraction time (run 4) for OHAE, whereas MAE has the highest TPC in run 1 with 1 g of solid solvent ratio and 45°C of extraction temperature. Run 6 had the lowest TPC for OHAE with the extraction conditions of 17.38 V/cm voltage and 8.7 min extraction time, and for MAE, the lowest TPC value was in run 5 with 6.55 g of solid solvent ratio and 34°C of extraction temperature. Also, for comparison with the traditional method, the TPC of avocado leaves was extracted with hot maceration conditions at 95°C and 120 min, and the TPC was obtained as 89.95 ± 0.05 mg GAE/100 g extract. These findings suggest that OHAE has the highest TPC than MAE and maceration. The extraction time for the highest TPC of OHAE, MAE, and maceration was in the order of 5.5, 5.0, and 120 min. The difference in extraction time between these techniques shows the advantages of phenolic recovery from avocado leaves in terms of time and energy savings by performing the extraction process in approximately 20.5 times less time. Therefore, OHAE can be a preferable alternative to MAE and traditional extraction methods, as it results in higher extract efficiency and TPC. In general, the results of the current study are also in line with the literature and showed that OHAE is a promising technology for the extraction of high‐value compounds with higher extraction efficiency, shorter extraction times, and lower solvent consumption (Kumar et al., 2014; Varghese et al., 2014). In addition, OHAE can be easily scaled up for industrial applications for the extraction of a wide range of compounds, including proteins, polyphenols, and other bioactive compounds (Varghese et al., 2014). This is the first study that compares the TPC of avocado leaves by OHAE and MAE applications; however, in the literature, there are different studies that conducted extraction by using different extraction methods (e.g., solvent and ultrasound‐assisted extraction). These studies obtained TPC values within the range of 3.41–92.850 mg GAE/100 g and reported in different studies such as 3.41 (Arukwe et al., 2012), 92.850 (Oboh et al., 2014), 270.73 (Kamagate et al., 2016), 23.710 (Yamassaki et al., 2017), 48.732 (Che‐Galicia et al., 2020), and 17.895 (mg GAE/100 g) (Abd Elkader et al., 2022).

In terms of TPC and extraction efficiency, the effect of OHAE application was better than MAE for the avocado leaves phenolics. The high total phenolic content and extraction efficiency obtained through ohmic extraction are likely due to the specific characteristics of this method, which uses an electric field to heat the sample quickly and uniformly. This method is known for its ability to extract bioactive compounds efficiency, which is reflected in the high total phenolic content and extraction efficiency obtained in this study (Weremfo et al., 2020). On the other hand, the low total phenolic content and relatively low extraction efficiency obtained through microwave extraction may be due to the limitations of this method, such as the lack of uniform heating and the potential for the degradation of bioactive compounds under high temperatures. It is possible that further optimization of the extraction conditions may be necessary to improve the efficiency of the MAE (Weremfo et al., 2020). Overall, these findings demonstrate the importance of choosing an appropriate extraction method for the bioactive compounds, as well as the need for optimization of extraction conditions to maximize the efficiency and total phenolic content of these compounds.

Table 2 shows the predictive models derived by fitting the second‐order polynomial model by multiple regression analysis of the experimental data via RSM, and the responses were verified for adequacy and fitness using analyses of variance (ANOVA) for both OHAE and MAE methods. According to the OHAE, the effects of the independent variables (*β*_1_ voltage gradient) and the first‐order term of time (*β*_2_) on the extract efficiency are statistically significant at the 99.9% level (*p* ≤ .001) in the model obtained through the response surface method. Among the second‐order variables in the model, the voltage value (*β*_11_) is statistically insignificant (*p* ≥ .05), while time (*β*_22_) is statistically significant (*p* ≤ .001). When examining the effect of the interaction between voltage and time (*β*_12_) on the extract efficiency, it was found to be statistically insignificant, indicating a high predictive power of the model for extract efficiency (*p* ≤ .001). The model explains 97.80% of the variations in extract efficiency that occur depending on the voltage and time parameters in the experimental design, indicating a high predictive power of the model for extract efficiency. Table 2 also displays the predictive models for MAE. The effect of temperature (*β*_1_), solid/solvent ratio (*β*_2_), and the second‐order variable of temperature (*β*_11_) on extraction efficiency were found to be statistically significant at the 99.9% level (*p* ≤ .001). Furthermore, the model's predicted extraction efficiency value based on the temperature and solid/solvent ratio parameters matched the experimental data quite closely (99%), indicating that the model has high predictive power in terms of extract efficiency.

**TABLE 2:** Regression coefficients of a polynomial function of a response surface for extraction efficiency (%) and TPC (mg GAE/100 g extract).

Model coefficients	Ohmic‐assisted extraction		Microwave‐assisted extraction	
	Efficiency	TPC	Efficiency	TPC
*β*_0_	36.19^b^	30648.2^b^	15.80^b^	10.6651^b^
*β*_1_	−0.4464^b^	−1887.9^b^	−0.9747^b^	−15.250^b^
*β*_2_	−0.7554^b^	352.5^b^	1.0498^b^	10.173^b^
*β*_11_	0.102^a^	−1553.5^b^	ns	12.331^b^
*β*_22_	−2.293^b^	−4174.8^b^	−0.2491^b^	5212^b^
*β*_12_	1.323^b^	−4879.2^b^	ns	15.759^b^
Model	^c^	^c^	^c^	^c^
Regression coefficient (*R* ^2^)	98.72	99.95	99.93	99.95
Adjusted *R* ^2^ (Adj‐*R* ^2^)	97.80	99.92	99.91	99.92
Predicted *R* ^2^ (Pred‐*R* ^2^)	95.49	99.82	99.84	99.75
Lack of fit	0.586	0.519	0.207	0.189

*Note*: For OHAE, *β*_1_: voltage gradient and *β*_2_: time. For MAE, *β*_1_: solid/solvent ratio and *β*_2_: temperature.

^a^ Statistically insignificant (*p* ≥ .05).

^b^ Statistically significant at the 99.9% level (*p* ≤ .001).

^c^ Statistically significant at the 99.9% level (*p* ≤ .001).

Table 2 also examined the significance of experimental factors involved in the OHAE and MAE of polyphenols from avocado leaves using ANOVA to evaluate the adequacy of the fitted model for TPC. This indicates that the voltage gradient (*β*_1_) has a significant effect on the TPC of the extracts obtained through OHAE (*p* ≤ .001), while the effect of extraction time (*β*_2_) is not significant (*p* ≥ .05). Moreover, the interaction between voltage gradient and extraction time, as well as the quadratic terms for both factors (*β*_11_, *β*_22_, and *β*_12_), have a significant effect on the TPC (*p* ≤ .001). Eventually, the predicted value of the TPC based on the voltage gradient and time parameters was found in good agreement with the data obtained in the experimental study (97.93%), and the model presented high predictive power in terms of TPC. In terms of MAE, both β1 (temperature) and *β*_2_ (solid/solvent ratio) were found to be statistically significant at the level of 99.9% (*p* ≤ .001) for the TPC. Among the second‐order variables in the model, the *β*_11_, *β*_22_, and their interaction (*β*_12_) were found to be statistically significant (*p* ≤ .001). Also, the experimental design explains the changes in the TPC of the extracts depending on the extraction parameters with a high prediction (99%). As a result, a strong correlation was found between the data obtained from the experiments and the values predicted by the models, suggesting that both models have the potential to accurately predict both TPC and extraction efficiency.

**FIGURE 1:**
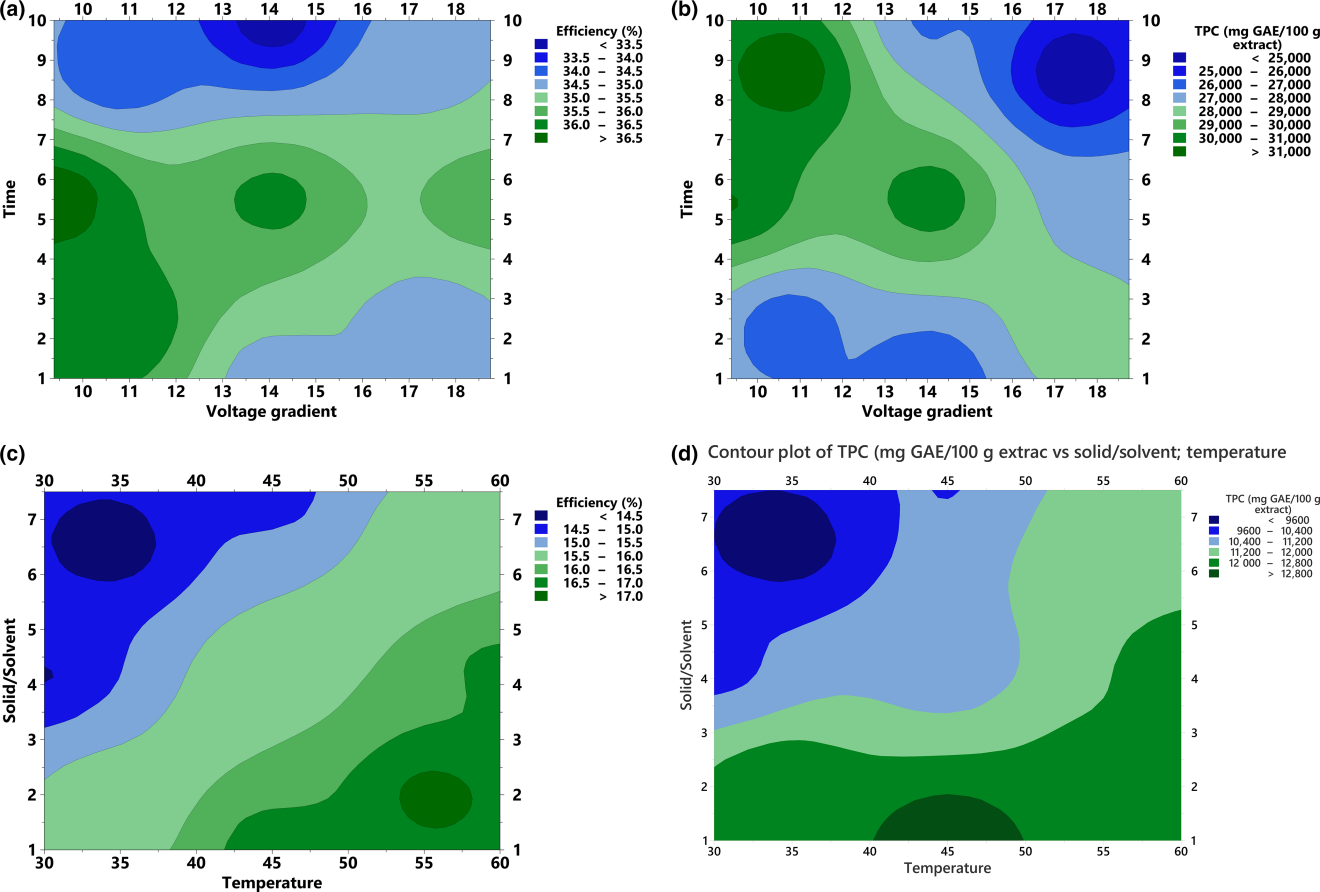
(a) Effects of voltage gradient (V/cm) and time (min) on TPC (mg GAE/100 g extract), (b) effects of voltage gradient (V/cm) and time (min) on extraction efficiency (%), (c) effects of temperature and solid/solvent ratio on extraction efficiency (%), and (d) effects of temperature and solid/solvent ratio on TPC (mg GAE/100 g extract).

The RSM interaction plots indicated the influence of independent variables (for OHAE, voltage gradient and time, and for MAE, temperature and solid solvent ratio) on the extraction efficiency and TPC (Figure 1). According to Figure 1a, the interaction between time and voltage gradient significantly affected extraction efficiency. High extraction efficiency was observed at low voltage values with longer extraction times. On the other hand, the shorter extraction time with a higher voltage value decreased the extraction efficiency. Under these conditions, the electric field applied to the leaves may charge the particles and causes the generation of heat. This may cause damage to the phenolics (degradation) as higher voltage gradient values can lead to faster heating and higher temperatures (Rodríguez et al., 2021). Khajehei and colleagues also reported that ohmic heating was a significant parameter for extraction efficiency due to its short processing time and faster heat production (Khajehei et al., 2017). The voltage gradient and time interaction were observed for their effect on the TPC in Figure 1b. It was shown that with an increase in the voltage gradient at the shorter extraction time, TPC increases up to a certain point and then partially decreased, whereas at longer extraction time with the rise of the voltage values, the TPC decreased. Furthermore, a lower voltage gradient with a longer extraction time causes an increase in the TPC, followed by a slight decrease. Both higher values for voltage gradient and extraction time increased the TPC at a certain point and then decreased. A recent study analyzed the effect of higher voltage and longer heating time on the phenolics from pineapple cores using ohmic heating (Gavahian & Chu, 2022). It was obtained that increasing the voltage up to 30 V resulted in a significant increase in phenolic extraction efficiency but increasing the voltage beyond 30 V did not result in further improvement in the extraction efficiency. Similar to the voltage gradient, the phenolic extraction efficiency increased for up to 30 min extraction and then started to decrease. It was concluded that the optimal voltage and time may depend on other factors such as the specific material being treated and the initial concentration of phenolics. Therefore, using RSM will provide a certain prediction for the extraction conditions to maximize extraction efficiency and TPC. A different study also reported the effect of a voltage gradient on phenolic release. It was found that the thermal effect of ohmic heating on grape leaves phenolics with the moderate electric field application caused a sudden release of polyphenolic compounds due to its effect on cell membranes (Jesus et al., 2020). Overall, the results of the study are consistent with the literature. The obtained results showed that the duration of the ohmic heating treatment and the amount of the voltage gradient can affect the extraction efficiency and total phenolic content. Longer treatment times can lead to higher extraction rates at lower voltage gradients, but increasing the amount of voltage gradient may further cause the degradation of phenolics (Rodríguez et al., 2021).

According to Figure 1c,d, the interaction between temperature and solid/solvent ratio significantly affected extraction efficiency and TPC for the MAE method. It was observed that the extraction efficiency decreased with an increase in the solid/solvent ratio at low temperatures, similarly, the efficiency again reduced at higher temperatures with a rise in the solid/solvent ratio. The reason for these results was illustrated in the literature that using a very high solid solvent ratio can decrease the extraction efficiency due to the limited availability of solvent for the extraction process (Gunalan et al., 2022). In addition, an increase in temperature can lead to a higher extraction efficiency due to the increased solubility and mobility of the phenolic compounds in the solvent. However, using a very high temperature can cause the degradation of the phenolic compounds, leading to a decrease in their concentration in the extracted solution (Oke et al., 2023). The extraction efficiency was only increased at lower solid/solvent ratios and high temperatures. Furthermore, Figure 1d indicates that TPC increased at higher temperatures and solid/solvent ratio but decreased at lower temperatures and higher solid/solvent ratio. However, the highest TFM was observed at lower temperatures and solid/solvent ratio. This is the first study that compares the effect of OHAE and MAE techniques on phenolics from Fuerte avocado leaves using RSM.

### 3.2 Optimization and validation of the optimum model conditions

Optimum extraction compositions for maximum extraction efficiency (%) and TPC (mg GAE/100 g extract) were obtained by central composite design for both OHAE and MAE methods. The optimum conditions of OHAE were determined as 9.38 V/cm of voltage gradient and 6 min extraction time. The model predicted that the extract efficiency would be in the range 35.99–36.97% and the TPC would be in the range11,015–11,886 mg GAE/100 g extract. To experimentally verify the predicted values of the model, avocado leaf samples were subjected to ohmic heating‐assisted extraction under the optimum conditions, and the extract efficiency and TPC were determined to be 36.86% and 11770.81 mg GAE/100 g extract, respectively. These values were within the predicted ranges of the model, indicating that the model was validated. Similarly, the optimum extraction conditions for MAE were detected as 60°C, 5 min, and 1 g dried leaf/100 mL water. The model predicted that the extraction efficiency would be in the range 17.52%–17.62% and the TPC would be in the range 132.80–135.31 mg GAE/100 g extract. The experiment was conducted according to the optimal conditions given above and the TPC was found to be 134.25 mg GAE/100 g extract and the extraction efficiency was determined to be 17.59%. These results showed that the experimental values of the optimal conditions agreed with the predicted value ranges given above.

### 3.3 Physicochemical properties of the extracts

#### 3.3.1 Phenolic composition

The phenolic composition of Fuerte avocado leaves phenolic water extract produced under optimum conditions using OHAE and MAE is given in Table 3. The OHAE extract (37.35 mg/100 g extract) had a greater amount of phenolics than the MAE extract (23.32 mg/100 g extract), but the phenolic composition of the MAE extract was found to be more diverse and abundant compared to the OHAE extract. The temperature applied during MAE application may cause phenolic degradation and therefore the amount of phenolics in the OHAE may be higher than in the MAE extract. The OHAE extract was analyzed for its phenolic compound composition, and the results indicated that the most abundant phenolic compound was epicatechin, followed by chlorogenic acid, rutin, protocatechuic acid, *p*‐hydroxybenzoic acid, and cinnamic acid, in decreasing order of concentration. On the other hand, the major phenolic of the MAE extract was detected as chlorogenic acid followed by epicatechin, *p*‐hydroxybenzoic acid, rosmarinic acid, quercetin, rutin, *p*‐coumaric acid, gallic acid, cinnamic acid, and protocatechuic acid. The phenolic composition and amounts in avocado leaves can vary depending on different factors such as solvent and extraction type, maturity and cultivar of leaves, temperature, and extraction time (Castro‐López et al., 2019; Che‐Galicia et al., 2020). On the other hand, the fast, efficient, and significant amount of phenolic extraction from avocado leaves by OHAE could be explained by not requiring high temperatures or strong solvents and providing the destruction of the cellular matrix through electrical application to enhance the greater release of phenolic compounds (Gavahian & Chu, 2022).

**TABLE 3:** Phenolic composition of optimum extract for OHAE and MAE techniques (mg/100 g extract).

Bioactive compounds	OHAE	MAE
Gallic acid	*	0.227 ± 0.000
Protocatechuic acid	3.129 ± 0.000	0.114 ± 0.001
*p*‐Hydroxybenzoic acid	0.407 ± 0.011	1.534 ± 0.032
Chlorogenic acid	10.964 ± 0.010	14.955 ± 0.011
Epicatechin	15.887 ± 0.050	3.466 ± 0.010
*p*‐Coumaric acid	*	0.341 ± 0.02
Rutin	6.837 ± 0.031	0.568 ± 0.010
Rosmarinic acid	*	1.068 ± 0.040
Cinnamic acid	0.129 ± 0.020	0.199 ± 0.001
Quercetin	*	0.852 ± 0.001
Total	37.35	23.32

*Note*: * Not detected.

The results also identify the effect of the extraction technique on the extraction of phenolics present in the leaves. According to Table 3, the results of the study indicate the superiority of MAE over OHAE for the extraction of gallic acid, p‐coumaric acid, rosmarinic acid, and quercetin. The explanation for this result may lie in the fact that higher levels of microwave temperature can cause more significant damage to the cellular structure which results in a different variety of phenolic compounds being released into the solvent (Routray & Orsat, 2014). The amount of these compounds is detected low with the MAE technique and due to the effect of electrochemical application, they may degrade in the OHAE application. The permeabilization of the cell membrane is expected to induce with OHAE application (Coelho et al., 2021). However, in the current study, dried avocado leaves, solid and poorly conductive, were used in the OHAE system to extract bioactive compounds. Therefore, it was required to apply higher electric fields which may lead to a degradation of some of the bioactive compounds in the leaves. In the literature, it is suggested that the degradation of these compounds may occur during the process due to the effects of heat and/or electrochemical degradation. Furthermore, the mechanisms and kinetic parameters of this degradation reaction can be impacted by the interaction between the electrode materials and the electrolysis products (Coelho et al., 2021). There are also some reports that explain the electrochemical degradation of gallic acid (Boye et al., 2006), *p*‐coumaric acid (Elaoud et al., 2012), rosmarinic acid (David et al., 2020), and quercetin (Zhou et al., 2007). In different studies, the phenolic composition of avocado leaves is also reported, for instance, Polat Kose et al. (2020) employed HPLC–MS to identify the phenolic compounds (mg/kg) in lyophilized water and ethanol extracts of dried avocado leaves. Their findings revealed the presence of caffeic acid (10.83–32.74 mg/kg), chlorogenic acid (28.83–852.81 mg/kg), and rutin (26.05–68.41 mg/kg). In another study, the phenolic compounds (mg/100 g) in avocado leaf water extract were quantified after utilization of methanol and HCl acid extraction as follows: shikimic acid (31.65), *p*‐hydroxybenzoic acid (2.51), ferulic acid (7.09), and epicatechin (2.84) by gas chromatography (Oboh et al., 2014). Yamassaki et al. (2017) analyzed the effect of different drying methods and storage conditions on the preservation of phenolics in avocado leaves. The study found that the primary phenolic compound in the avocado leaves was chlorogenic acid, followed by catechin, epicatechin, procyanidin B2, and rutin (Yamassaki et al., 2017). A more recent report analyzed the phenolic content of leaves from seven Mexican avocado cultivars which were ethanolic extracted using UAE. The phenolic composition was detected LC–MS (Castro‐López et al., 2019). All cultivars' predominant phenolic compound (mg/g extract) was detected as chlorogenic acid ranging from 40.8 to 63.9. The amounts of other abundant phenolic compounds (mg/g extract) were varying between 2.2 and 12.7 for epicatechin, 2.2 and 6.1 for catechin, 1.4 and 4.7 for gallic acid, 1.3 and 3.1 for caffeic acid, and 0.4 and 1.4 for *p*‐coumaric acid, respectively. The results of the current study are mostly in agreement with the previous reports. A comparison of these research findings with existing literature suggests that the phenolic compound levels in avocado leaf extracts may indicate variations due to factors such as the avocado variety used, differences in the solvent and extraction method, climatic growth conditions, and harvest time.

#### 3.3.2 Antioxidant and antidiabetic activities

The rich phenolic content of avocado leaves may promote health. In several studies, avocado leaves phenolics were reported for their antidiabetic, lipid‐lowering, cardioprotective, antithrombotic, antiobesity, antiatherosclerotic, and antihypertensive effects due to their bioactive compound contents (Abd Elkader et al., 2022). The antidiabetic and antioxidant activities of Fuerte avocado leaves' optimized extracts by OHAE and MAE methods were given in Table 4. The IC_50_ (mg/mL) values of OHAE and MAE applied extracts for inhibition of α‐glucosidase activity were detected as 0.85 and 1.14, respectively. As a positive control, a commercial drug acarbose was tested for its inhibition effect on α‐glucosidase enzyme, and the IC_50_ value was found as 0.96 ± 0.05 mg/mL. These results are in harmony with those reported by a recent study about avocado leaf methanolic extract exhibited significant inhibition of α‐glucosidase enzymes, with an IC_50_ value of 0.69 mg/mL, whereas the positive control glibenclamide's IC_50_ value was reported as 1.45 mg/mL (Ehikioya et al., 2023). The highest inhibitory activity against α‐glucosidase was with the OHAE extract compared to acarbose and MAE extract, respectively. Our results are shedding light on understanding the vital role of extraction methods on antidiabetic activity and agree with a recent study by Zeleke et al. (2023).

**TABLE 4:** Antioxidant and antidiabetic activities of Fuerte avocado leaves.

Functional properties	OHAE	MAE
DPPH (IC_50_ mg/mL)	2.96 ± 0.15^x^	3.41 ± 0.24^y^
α‐Glucosidase activity (IC_50_ mg/mL)	0.85 ± 0.04^x^	1.14 ± 0.01^y^

*Note*: Means that are shared by different superscripts within each row indicate a Tukey's test comparison between the extracts at *p* < .05.

Recently, many polyphenolic compounds have been detected in plants such as epicatechin, quercetin, chlorogenic acid, and rutin, which were reported as potent inhibitors of glucosidase enzymes (Chokki et al., 2020). As both extracts are rich in these bioactive compounds, their potential glucosidase inhibitory activity may be attributed to the synergistic activity of both the major and minor bioactive metabolites.

The presence of higher levels of reactive oxygen species and free radicals is responsible for oxidative stress, a condition that poses a significant risk to human health and can lead to various illnesses like cancer, aging, cytotoxicity, liver damage, and cardiovascular diseases, and assessment of the antioxidant potential of different compounds by the DPPH radical scavenging activity is commonly employed (Gao et al., 2022). The DPPH radicals scavenging activity (IC_50_ mg/mL) of OHAE and MAE were detected as 2.96 and 3.41, respectively (Table 4). Different studies have reported the antioxidant activity of phenolic compounds in avocado leaves. For instance, Oboh et al. (2016) analyzed the water extract of avocado leaves by maceration and reported the IC_50_ value as 25.21 mg/mL for DPPH radical scavenging activity (Oboh et al., 2016). The current study result's antioxidant activity was higher than this study and this could be explained by the impact of OHAE and MAE application for the phenolic extraction of avocado leaves. Moreover, Castro‐López et al. (2019) reported an IC_50_ value of 0.272 mg/mL for the free radical scavenging activity of ethanolic extract obtained by ultrasound‐assisted extraction (UAE) of the Platano Delgado variety of avocado leaves (Castro‐López et al., 2019). According to the literature, ethanolic extraction of leaves displays higher antioxidant activity than its aqueous extraction (Tonfack Djikeng et al., 2018). The differences in the results may be attributed to factors such as the different solvents and methods used in the extraction process, and variations in the avocado cultivars.

## 4 CONCLUSION

The utilization of OHAE and MAE as a contemporary method of extraction offers several benefits over traditional extraction techniques, including reduced solvent consumption and extraction time. As a result, it is a valuable approach for obtaining bioactive phytochemicals. This study optimized the extraction of phenolic compounds from pruning waste of Fuerte avocado leaves using CCD with both OHAE and MAE techniques for the first time. The developed models showed high reliability, and the optimal OHAE and MAE conditions were found to be 9.38 V/cm voltage gradient and 6 min extraction time (desirability = 0.9309), and 60°C, 5 min, and 1 g dried leaf/100 mL water (desirability = 0.9940), respectively. The extracts obtained through both techniques were rich in polyphenols with high antioxidant activity, with OHAE showing higher results in terms of TPC, DPPH, and antidiabetic activities. MAE extracts exhibited more phenolic compounds than OHAE extracts and were quantified by HPLC‐DAD. The major phenolic of OHAE was detected as epicatechin while for MAE it was chlorogenic acid. The results of the study indicate the superiority of MAE over OHAE for the extraction of gallic acid, *p*‐coumaric acid, rosmarinic acid, and quercetin. This outcome may be attributed to the volumetric and selective heating mechanism of microwaves, which causes a rapid increase in temperature and internal pressure, ultimately resulting in the breakdown of the sample surface and the release of various types of phenolic compounds. In comparison, OHAE is not requiring high temperatures or strong solvents and provides the destruction of the cellular matrix through electrical application to enhance the greater release of phenolic compounds. Overall, it was found that ohmic heating is a highly effective system for TPC and extract efficiency by overcoming the disadvantages inherent in traditional heating methods by using heat generation from inside to outside. In conclusion, the findings suggest that the phenolic compounds extracted from avocado leaves have the potential to support the management of diabetes, as evidenced by their superior ability to inhibit α‐glucosidase enzymes compared to other extracts. Moreover, these findings may contribute to the promotion of a circular economy by facilitating the valorization of the examined pruning waste material.

## AUTHOR CONTRIBUTIONS

**LALE GUMUSTEPE:** Conceptualization (equal); formal analysis (equal); investigation (equal); methodology (equal); writing – original draft (supporting). **NEVRIYE KURT:** Conceptualization (equal); formal analysis (equal); investigation (equal); methodology (equal); writing – original draft (supporting). **EBRU Aydın:** Conceptualization (equal); formal analysis (equal); funding acquisition (lead); methodology (equal); project administration (lead); supervision (lead); writing – original draft (equal); writing – review and editing (equal). **Gulcan Ozkan:** Conceptualization (equal); formal analysis (equal); investigation (equal); methodology (equal); supervision (supporting); writing – original draft (equal); writing – review and editing (equal).

## CONFLICT OF INTEREST STATEMENT

We declare that there are no conflicts of interest associated with this manuscript.

## Data Availability

Data are available on request from the authors.
